# Soluble C-Type Lectin-Receptor Ligands Stimulate ROS Production in Dendritic Cells and Potentiate Killing of MRSA as Well as the MRSA Induced IL-12 Production

**DOI:** 10.3389/fimmu.2022.845881

**Published:** 2022-03-21

**Authors:** Helene M. S. Eld, Peter R. Johnsen, Emilie M. Nielsen, Frederikke Z. Jørgensen, Marie Lindstrøm-Svendsen, Mara Baldry, Hanne Ingmer, Hanne Frøkiær

**Affiliations:** Department of Veterinary and Animal Sciences, University of Copenhagen, Frederiksberg, Denmark

**Keywords:** dendritic cells, mannose receptor, dectin-1, IL-12 potentiating, IFN-beta potentiating, endosomal killing

## Abstract

Methicillin resistant *Staphylococcus aureus* (MRSA) has developed resistance to most β-lactam antibiotics leaving few treatment options against infections with MRSA. Through mannose receptors, mannan potentiates IL-12 production induced by Gram-positive bacteria, a cytokine crucial in the clearance of *S. aureus* infection. We investigated the IL-12 potentiating effect of mannan pre-treatment of bone marrow-derived dendritic cells prior to stimulation with clinical MRSA strains. Mannan almost doubled IL-12 as well as IFN-β production in response to USA300, also when USA300 was treated with the β-lactam cefoxitin. The MRSA-induced IL-12 production was dependent on bacterial uptake and reactive oxygen species (ROS). Mannan alone induced ROS production, and in combination with USA300, the ROS produced corresponded to the sum induced by mannan and USA300. Addition of a monoclonal antibody against the mannose receptor likewise enhanced USA300-induced IL-12 and induced ROS production. Mannan addition further increased the endocytosis as well as the rate of endosomal killing of bacteria. Pre-treatment with soluble β-glucans also induced ROS and potentiated the USA300-induced IL-12 indicating that other C-type receptors may play a similar role. In the presence of the pro-inflammatory mediators, GM-CSF or IFN-γ, the mannan-enhanced IL-12 production increased further. The USA300-induced and the mannan-facilitated enhanced IFN-β and IL-12 showed same dependency on MAPK c-Jun N-terminal kinase signaling, suggesting that mannan enhances the signals already induced by the bacteria, rather than changing them. We suggest that the C-type lectin-induced ROS production is a key factor in the IFN-β and IL-12 potentiation.

## Introduction

*Staphylococcus aureus* is an opportunistic pathogen able to cause severe infections in skin and soft tissue and is the most common cause of bacteremia (1). In order for the immune system to combat the bacterium, *S. aureus* must be endocytosed and degraded by macrophages and dendritic cells to induce an efficient immune response. To this end, the induction of interleukin (IL)-12 has been proven indispensable for the eradication of the bacterium (2, 3). *S. aureus*, however, has proven quite resistant towards endosomal degradation in macrophages and dendritic cells resulting in its evasion and spread in the body. Moreover, *S.aureu*s is a rather poor inducer of IL-12 (4).

Dendritic cells, especially, are potent producers of IL-12 in response to bacteria and viruses (5). The production of IL-12 directs T helper 1 (Th1) polarization in naïve T cells and activation of NK-cells leading to the production of interferon (IFN)-γ, in turn enhancing the elimination of bacteria (6, 7). Production of IL-12 induced by Gram-positive bacteria depends on uptake and degradation of the bacteria in order to facilitate endosomal activation of Toll-like receptors (TLRs) (8–11). Furthermore, production of reactive oxidative species (ROS) seems to be an important event preceding the IL-12 induction by *Lactobacillus acidophilus*, suggesting this is also the case for *S. aureus* (12, 13).

In order to target pathogens, dendritic cells express pathogen recognition receptors (PRR) recognizing conserved structures, the so-called pathogen associated molecular patterns. Among the PRRs, a group of C-type lectin receptors including mannose receptor (MR) and dendritic cell-specific intercellular adhesion molecule-3-grabbing non-integrin (DC-SIGN) recognize mannose-containing structures. In addition to be a highly efficient samplers of mannose-containing antigens, these receptors are reported to hold immune modulatory effects (14, 15). MR’s involvement in phagocytosis of bacteria is controversial but a recent study showed that MR is involved in the phagocytosis of *M. tuberculosis* (16). We have shown that mannan through MR increases phagocytosis of *L. acidophilus* without being directly involved as a phagocytic receptor (13). Mannan added to bone marrow derived dendritic cells (DCs) stimulated with the Gram-positive bacteria *L. acidophilus* and *S. aureus*, but not with the Gram-negative bacterium *E. coli*, led to increased IL-12 production. Furthermore, mannan was able to up-regulate SLAMF1 (CD150) expression in DCs and to enhance the ROS production in *L. acidophilus*-stimulated DCs (13). Likewise, activation of the β-glucan receptor dectin-1 was shown to induce ROS production and the ability to potentiate an inflammatory response against pathogens (17, 18). We have previously demonstrated that soluble but not aggregated dispensed β-glucan enhances the *L. acidophilus* induced IL-12 response (19). Hence, ligation of several C-type receptors may prove to modulate the cytokine response induced by pathogens.

Because mannan enhances bacterial uptake, ROS production, endosomal degradation, and IL-12 production in DCs in response to *L. acidophilus*, we hypothesized that mannan would exhibit the same effects in DCs in response to *S.aureus*, including clinical methilicin resistant *S.aureus* (MRSA) strains such as USA300. We investigated the intracellular signaling pathways leading to the induced IL-12 production in MRSA-stimulated DCs pre-treated with mannan, and whether inflammatory conditions or β-lactam treatment of MRSA altered these effects. Furthermore, we investigated whether only mannan or other soluble ligands activating clathrin-dependent C-type lectin receptors could induce ROS and IL-12 production, important for efficient bacterial clearance.

Potentiation of the IL-12 induction through soluble carbohydrates represents a simple strategy to strengthen the immune defense towards MRSA also in combination with antibiotics treatment. In the present study we found that mannan as well as soluble β-glucan in the absence of bacteria increased ROS production in DCs and that pretreatment of DCs with these C-type lectin ligands represents a simple way to enhance the phagocytosis and killing of *S. aureus* and to induce a strong IL-12 response imperative for the clearance of *S. aureus*.

## Methods

### Generation of Bone Marrow-Derived Dendritic Cells and Macrophages

DCs were generated using bone marrow cells from C57BL/6NTac mice as previously described (20). In brief, cells were isolated from cleansed tibia and femur by flushing with cold PBS. The isolated cells were seeded at 3·10^5^ cells/ml in RPMI 1640 medium (Thermo Fisher Scientific, Waltham, MA, USA) containing 10% (v/v) FCS, 4mM L-glutamine, penicillin (100 U/ml) and streptomycin (100 U/ml), and 50 μM β-mercaptoethanol (all products from Thermo Fisher Scientific). Complete medium was supplemented with 15 ng/ml granulocyte-macrophage colony stimulating factors (GM-CSF) for dendritic cell differentiation. For macrophage differentiation, isolated cells were seeded at 4·10^5^ cells/ml in complete medium supplemented with 30 ng/ml macrophage (M)-CSF. On day three and six new medium with GM-CSF or M-CSF was added, while on day eight the DCs were harvested by collecting all non-bound cells and for macrophages, cells were harvested by collecting all surface-bound cells using a cell lifter (Thermo Fisher Scientific).

### Bacterial Strains

The clinical methicillin-resistant *S. aureus* strain USA300 was used. Two isogenic MRSA strains, SA1 (A9781) and SA2 (A9788) were also included. Bacteria were streaked on tryptic soy agar (TSA) and after ~16 h of incubation (37°C), a single colony was inoculated and grown in tryptic soy broth (TSB) overnight. Bacteria were then re-inoculated (OD_600_: 0.05) in TSB with or without 2 µg/ml cefoxitin to assess the effects of β-lactam and grown to exponential phase (OD_600_: 0.7-0.8). Bacteria were washed twice in PBS and plated for CFU determination and hereafter they were UV-treated using pulsed UV radiation for a minimum of 90 seconds (6 s/pulse with monochromatic wavelength of 254 nm; CL-1000 crosslinker; UVP, Cambridge, United Kingdom). After UV-treatment, bacteria were plated to confirm no viability.

### Cell Stimulations and Treatments

DCs (2·10^6^ cells/ml) were stimulated with UV-treated USA300 at MOI 10, unless otherwise stated, and incubated at 37°C in 5% CO_2_ at indicated hours. DCs were treated with 100 µg/ml mannan from *Saccharomyces cerevisiae* (Sigma-Aldrich, St. Louis, MO, USA) 30 min prior to bacteria stimulation. Prior to bacterial stimulation BMDCs were pre-treated with 0.5 µg/ml cytochalasin D (CytD) (Sigma-Aldrich) for 1 h to inhibit bacterial uptake, with 300 µM apocynin (R&D systems, Minneapolis, MN, USA) for 30 min to inhibit assembly of the NADPH oxidase and ROS production and with 50 nM bafilomycin A1 (BafA) (Sigma-Aldrich) or 40 mM NH_4_Cl (Sigma-Aldrich) for 1 h to inhibit phagosome acidification. MAPK pathways were inhibited in DCs by pre-treating for 1 h with 25 µM JNK inhibitor (Sigma-Aldrich, SP600125), 10 µM p38 inhibitor (Sigma-Aldrich, SB203580) or 10 µM MEK 1/2 inhibitor (Sigma-Aldrich, UO126). Factors present during inflammation such as GM-CSF (15 ng/ml) or IFN-γ (1 ng/ml, Thermo Fisher Scientific) was added to the medium of DCs just prior to bacterial stimulation.

### Cytokine Production by DCs

Cytokine secretion from DCs was measured in supernatants after 20 hours of bacterial stimulation, unless other is stated, using duoset™ ELISA for murine IL-12p70 (DY419), IFN-β (DY814), IL-10 (DY417) and IL-1β (DY401) from R&D systems according to manufacturer’s instructions.

### RNA Extraction, cDNA Synthesis and Quantitative Real-Time PCR

Using the MagMAX-96 total RNA Isolation Kit (Applied Biosystems, Foster City, CA, USA), mRNA was extracted from DCs according to manufacturer’s instructions at different time points. RNA (500 ng) extracted from each sample was converted to cDNA using Applied Biosystems™ High-Capacity cDNA Reverse Transcription Kit. Cycle threshold (Ct) value of *Actb* (β-actin) was used as reference gene to define ΔCt for each sample (ΔCt_target_=Ct_target_-Ct_reference_). The ΔΔCt method was used to estimate fold-change expression of each gene. First, ΔCt of the control sample (un-stimulated BMDCs) from the respective time point is subtracted from the ΔCt of a target sample (ΔΔCt=ΔCt_target_- ΔCt_control_). Fold-change in gene expression was defined by 2^-ΔΔCt^.

### Endocytosis of AF-647-Conjugated USA300

Bacterial uptake was assessed using flow cytometry with USA300 conjugated with Alexa Flour-647 (AF-647) (Molecular Probes, Eugene, OR, USA). DCs (2·10^6^ cells/ml) were incubated with AF-647-conjugated USA300 at MOI 50 for 1 h, hereafter cells were washed twice (PBS, 1% FCS) and fixated in 1% formaldehyde. The fluorescence of APC was measured in DCs using FACS BD canto II flow cytometer (BD biosciences, Franklin Lakes, NJ, USA). As a negative control, DCs were pre-treated with CytD (0.5 μg/ml) for 1 h to block the uptake of bacteria.

### Measurement of ROS Production in DCs

DCs (2·10^6^ cells/ml) were incubated with 5 µM carboxy-H_2_DCFDA (Thermo Fisher Scientific) and pre-treated or not with 100 µg/ml mannan for 30 min at 37°C, 5% CO_2_. In some experiments, GM-CSF (15 ng/ml) or IFN-γ (1 ng/ml) was added to the medium of the cells, prior to mannan stimulation. DCs were then stimulated with USA300 at MOI 10 for 4 h. Oxidation of internalized carboxy-H_2_DCF (converted from carboxy-H_2_DCFDA by intracellular esterases) to carboxy-DCF was quantified using flow cytometry (BD Canto II, BD Biosciences) by measuring the intensity FITC. Mean fluorescence intensity (MFI) of DCs was used to compare ROS production between samples.

### Endosomal Killing of Bacteria

Bone marrow derived macrophages were seeded at 6.7·10^5^ cells/ml in complete medium without antibiotics a day prior to addition of bacteria, for cells to adhere to the bottom of the well. Cells were pre-treated with mannan (100 µg/ml) or not for 30 min before infecting with USA300 at MOI 50 for 1 h at 37°C, 5% CO_2_. After two times washing with PBS, cells were incubated 1 h with medium containing 100 μg/ml gentamicin (Sigma-Aldrich) to kill extracellular bacteria. After washing, the cells either further incubated in medium with 5 μg/ml gentamicin for 3 h or 20 h or directly lysed with sterile dH_2_O for 30 min at room temperature, releasing intracellular bacteria from the cells. These samples were referred to as 0 h. At 3 h and 20 h, samples were washed and lysed with dH_2_O as described above. The number of intracellular bacteria was assessed by counting CFU from plated cell lysates. For each group (cells with or without mannan pre-treatment), CFU/ml of intracellular bacteria for each time point were normalized to the initial intracellular bacteria (0 h CFU/ml) and are shown as percentages.

## Results

### Mannan Increases the IL-12 Production in MRSA-Stimulated DCs

**Figure 1:**
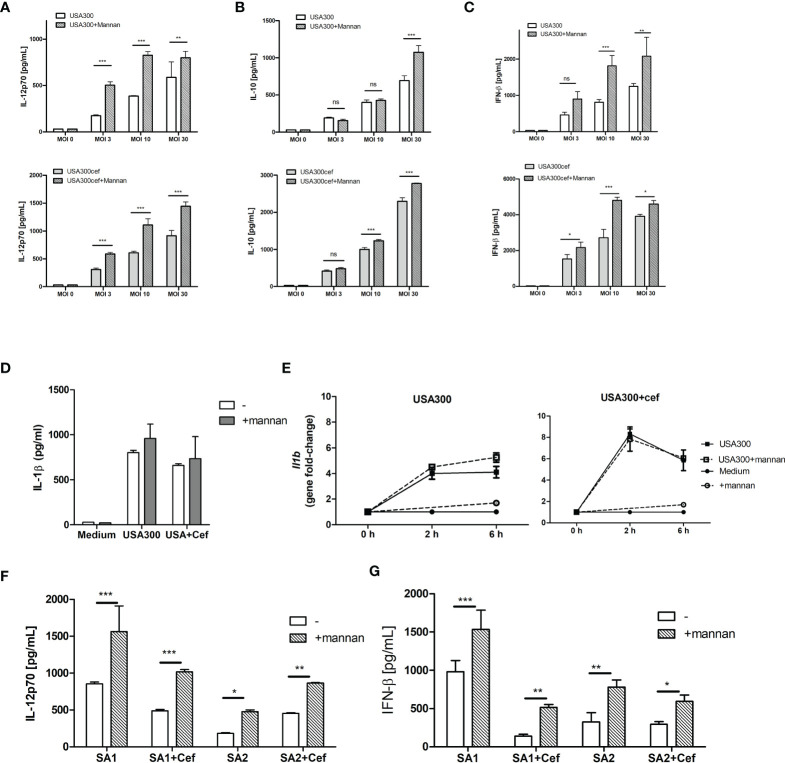
Mannan increases IL-12 and INF-β production in USA300-stimulated DCs. DCs were pre-treated or not with mannan (100 µg/ml) 30 min prior to infecting with UV-treated USA300 or cefoxitin-grown USA300 at MOI 3, 10 and 30. IL-12 **(A)**, IL-10 **(B)**, IFN-β **(C)** were measured in supernatant by ELISA, n=3 (SD) wells for each treatment. IL-1β production after stimulation with MOI 10 **(D)**, and *Il1b* expression at 0 h, 2 h and 6 h (error bars based on two technical replicates) **(E)**. DCs were pre-treated or not with mannan 30 min prior to stimulation with the clinical MRSA SA1 and SA2 grown with or without cefoxitin, IL-12 and IFN-β was measured in supernatant after 20 h **(F, G)**, n=3, error bars: SD. 2-way ANOVA was performed, *p < 0.05, **p < 0.01, ***p < 0.001. ns, non-significant. All data shown are representative data of one out of at least 3 independent experiments.

To investigate whether mannan affected IL-12 production in DCs exposed to MRSA, DCs were pre-treated with mannan 30 min prior to stimulation with increasing MOIs of the MRSA strain USA300 grown with or without the β-lactam cefoxitin. We have previously found that a concentration of 100 µg/ml mannan is the optimal stimulation (13). Stimulating DCs with USA300 or cefoxitin-treated USA300 resulted in dose-dependent IL-12, IL-10 and IFN-β responses (**Figures 1A–C**). Mannan alone did not induce cytokine production but pre-treating with mannan prior to bacterial stimulation increased the production of IL-12 and INF-β by up to 100% (**Figures 1A, C**). In contrast, IL-10 production was only slightly increased (20-30%) by mannan pretreatment and only at high MOI (**Figure 1B**). Mannan pre-treatment did not increase the secreted IL-1β in the supernatant (**Figure 1D**), nor the IL-1β expression, after stimulation with USA300 or cefoxitin-treated USA300 (**Figure 1E**). To see if mannan exhibited same effect on the response to other MRSA strains, DCs were pre-treated with mannan prior to stimulation with two other clinical MRSA strains, SA1 and SA2, previously shown to possess different phenotypical characteristics (21). Similarly to USA300, both strains showed enhanced IL-12 and IFN-β induction upon pre-treatment with mannan (**Figures 1F, G**).

**Figure 2:**
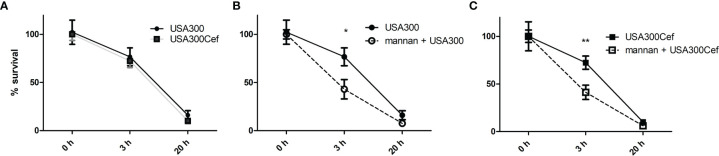
Mannan increases the rate of endosomal killing of USA300. DCs were infected for 1 h with live USA300 **(A, B)** or cefoxitin-grown USA300 **(A, C)** at MOI 50. After 1 h of gentamicin (t = 0), DCs were incubated for 0, 3, and 20 h before cells were washed and lysed. CFU was counted from plated cell lysates and are shown as percentages compared to initial CFU (t = 0 h). Prior to addition of bacteria, cells were pre-treated or not **(B, C)** with mannan for 30 min. Data [average (SEM)] based on four pooled separate experiments. T-test, *p < 0.05, **p < 0.01.

Intracellular killing of bacteria by dendritic cell and macrophages is important for the response against bacteria and clearance of infection. Mannan was previous shown to accelerate endosomal killing of the commensal *L. acidophilus* NCFM (13). To see whether mannan pre-treatment affected the killing of the endocytosed USA300, macrophages were infected with live untreated or cefoxitin-treated USA300 and the number of intracellular bacteria recovered at 0, 3 and 20 h of incubation was assessed. Cefoxitin treatment did not lead to change in the number of bacteria recovered at any time point (**Figure 2A**). Independently of cefoxitin-treatment, pre-treatment with mannan accelerated the intracellular killing of USA300 in macrophages resulting in a reduced number of live bacteria at 3h, however at 20h the recovered numbers were comparable (**Figures 2B, C**).

Taken together, although mannan *per se* did not induce cytokine production in DCs, mannan pretreatment clearly enhanced the IL-12 and IFN-β production induced by MRSA and cefoxitin treated MRSA and led a more prompt intracellular killing in macrophages.

### Mannan *per se* Induces ROS Production and Weak Maturation of DCs

**Figure 3:**
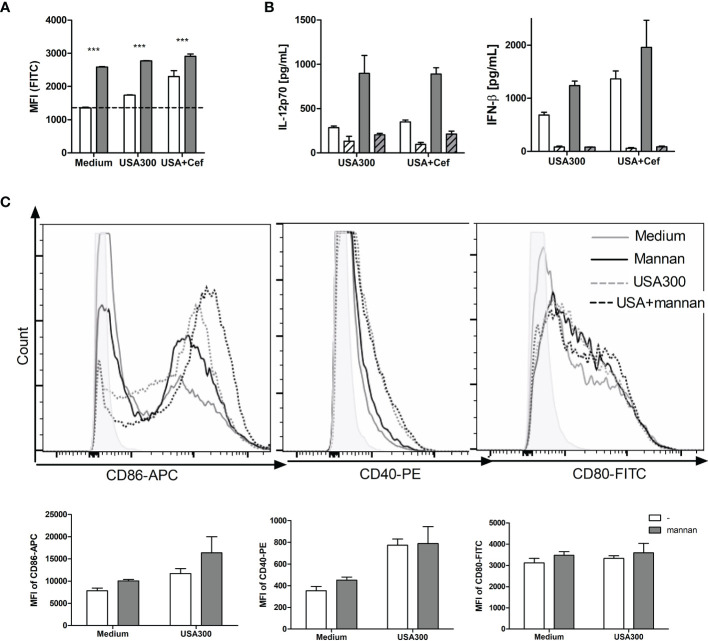
*Mannan per se induces ROS production and weak maturation of DCs.* DCs were pre-treated or not with mannan and left unstimulated or stimulated with USA300 or cefoxitin-grown USA300 at MOI 10. The production of ROS after 4 h measured as cellular oxidation of carboxy-H_2_DCF and shown as mean fluorescence intensity (MFI) of the cells ANOVA: ***p < 0.01 **(A)**. White bars: Samples without mannan pre-treatment; black bars: samples pre-treated with mannan. The IL-12 and IFN-β production in cells stimulated with USA300 or cefoxitin-grown USA300 with or without pre-treatment with mannan **(B)**. The concentration of IL-12 and IFN-β in the supernatant 20 h after stimulation with USA300 or cefoxitin-grown USA-300 (white bars), with mannan pre-treatment (black bars), and with initial treatment of cells with apocynin (hatched bars). All data are based on three separately treated culture wells and results are representative of two or three experiments. **(C)** Histograms showing the expression of CD86, CD80 and CD40 after treatment with mannan and/or stimulation with USA300. Bar plots of the same data show MFI of three culture samples of each treatment.

To investigate how pre-treatment with mannan leads to enhanced IL-12 and IFN-β production and endosomal killing, we speculated that mannan may induce or enhance the USA300-induced ROS production in DCs measured as oxidation of internalized carboxy-H_2_DCF to the fluorescent compound carboxy-DCF. Mannan alone induced a ROS production in DCs that was almost three times the magnitude of the production induced by USA300, and the induction by mannan and USA300 together corresponded to the sum of induction by the two stimuli (**Figure 3A**). Cefoxitin treatment of USA300 increased the ROS production but the increase upon mannan pretreatment was less compared to stimulation with untreated USA300 (**Figure 3A**). To investigate whether inhibition of the ROS production affected IL-12 production, DCs were incubated with the NADPH oxidase inhibitor apocynin (22) for 30 min prior to mannan pretreatment and bacterial stimulation. Apocynin fully abrogated the IFN-β induction and reduced the IL-12 production induced by USA300 by approximately 50% and, independently of cefoxitin, the IL-12 production induced by mannan and USA300 was reduced to a comparable level (**Figure 3B**). Mannan treatment alone (black line) slightly upregulated the co-stimulatory molecule CD86, and mannan pre-treatment also slightly enhanced the CD86 expression induced by USA300 (black dotted line) compared to USA300 alone (grey dotted line). Mannan showed a similar but much weaker effect on the CD80 expression (**Figure 3C**). Mannan alone or as pre-treatment did not affect the CD40 expression on BMDCs, while stimulation with USA300 induced a strong increase in CD40 expression (**Figure 3C**). Taken together, mannan is a potent inducer of ROS formation and may lead to some increase of CD86 and CD80 but not of CD40 expression. The induction of IFN-β appears to be fully and IL-12 partly dependent on NADH oxidase activation.

### Mannan-Enhanced IL-12 Production Depends on Clathrin-Coated Pits and on β-Actin-Mediated Endocytosis of *S. aureus*

**Figure 4:**
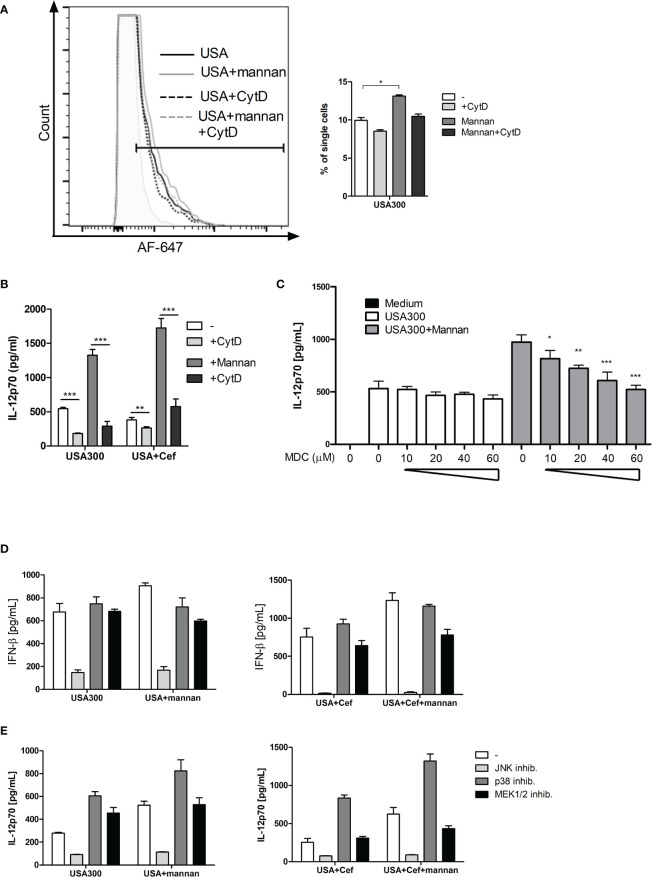
*The IL-12-enhancing effect of mannan is dependent on bacteria uptake and is abrogated by inhibition of clathrin-coated pits.* DCs were pre-treated with CytD and/or mannan and infected with AF-647-labeled USA300 at MOI 50 for 1 h. Fluorescence intensity was measured by flow cytometry. Histogram shows a representative example of AF-647-USA300 uptake. Bar graph shows percentages of DCs taking up bacteria, paired t-test was performed on samples subtracted their respective CytD-treated samples **(A)**. DCs were treated with CytD for 1 h and treated with mannan 30 min prior to infection with USA300. Cytokines were measured in supernatant by ELISA **(B)**. DCs were treated with increasing concentration of monodansylcadaverine (MDC) and pre-treated (black bars) or not (white bars) with mannan prior to stimulation with USA300 MOI 10. IL-12 in the supernatant was measured by ELISA **(C)**. The effect of inhibition of MAP kinases on the IFN-β **(D)** and IL-12 **(E)** concentration in cell supernatants after USA300 stimulation with or without mannan pre-treatment. All data are based on three separately stimulated replicates and are representative of two or more experiments. *p < 0.05, **p < 0.01, ***p < 0.001.

To see whether mannan increased the uptake of USA300, DCs were incubated with AF647-labeled USA300. As visualized by flow cytometry, the labeled bacteria were taken up by the cells and mannan increased the number of cells that had taken up the labeled USA300 (**Figure 4A**). Inhibition of bacterial uptake by addition of cytochalasin D, inhibited the IL-12 production induced by USA300 with or without mannan pre-treatment (**Figure 4B**).

Upon binding of mannan to the mannose receptor, the receptor is internalized by clathrin-coated pits. To investigate whether clathrin-coated pits activity was important for the mannan-enhanced IL-12 production, DCs were treated with monodansylcadaverine (MDC) at increasing concentrations (10-60 µM) prior to mannan pretreatment and USA300 stimulation. MDC did not affect the IL-12 production induced by USA300 (**Figure 4C**). In contrast, MDC as low as 10 µM decreased the mannan-enhanced IL-12 in USA300-stimulated DCs and increasing concentrations of MDC further reinforced the inhibition of IL-12 production resulting in a full inhibition of the mannan-enhanced IL-12 production at 60 µM MDC. To investigate whether mannan changed the MAP kinase pathways involved in INF-β and IL-12 production in response to USA300 (20, 23), DCs were pre-treated with inhibitors for each of the MAP kinases JNK, p38 or ERK1/2 for 1 h prior to addition of mannan and subsequent stimulation with USA300. Inhibition of JNK completely abrogated the USA300-induced IFN-β regardless of mannan pre-treatment and cefoxitin treatment of MRSA while inhibition of p38 or ERK1/2 resulted in reduction of the mannan-induced enhancement of INF-β (**Figure 4D**). IL-12 production was also abrogated by inhibition of JNK, while inhibition of p38 led to an increased IL-12 induction both with and without pre-stimulation with mannan. Of note, inhibiting ERK1/2 led to an increase in IL-12 by USA300, but not if pre-treated with mannan. Conversely, if stimulated with cefoxitin-treated USA300 inhibition of ERK1/2 did not affect IL-12 induction; only if pre-treated with mannan there was a decrease in IL-12 (**Figure 4E**). Hence, in a clathrin-dependent way mannan leads to enhanced β-actin-dependent uptake of *S. aureus* but does not induce major changes in the signaling pathways involved in induction of INF-β and IL-12.

### The IL-12 Enhancement by Mannan Is Not Affected by Inflammatory Conditions

**Figure 5:**
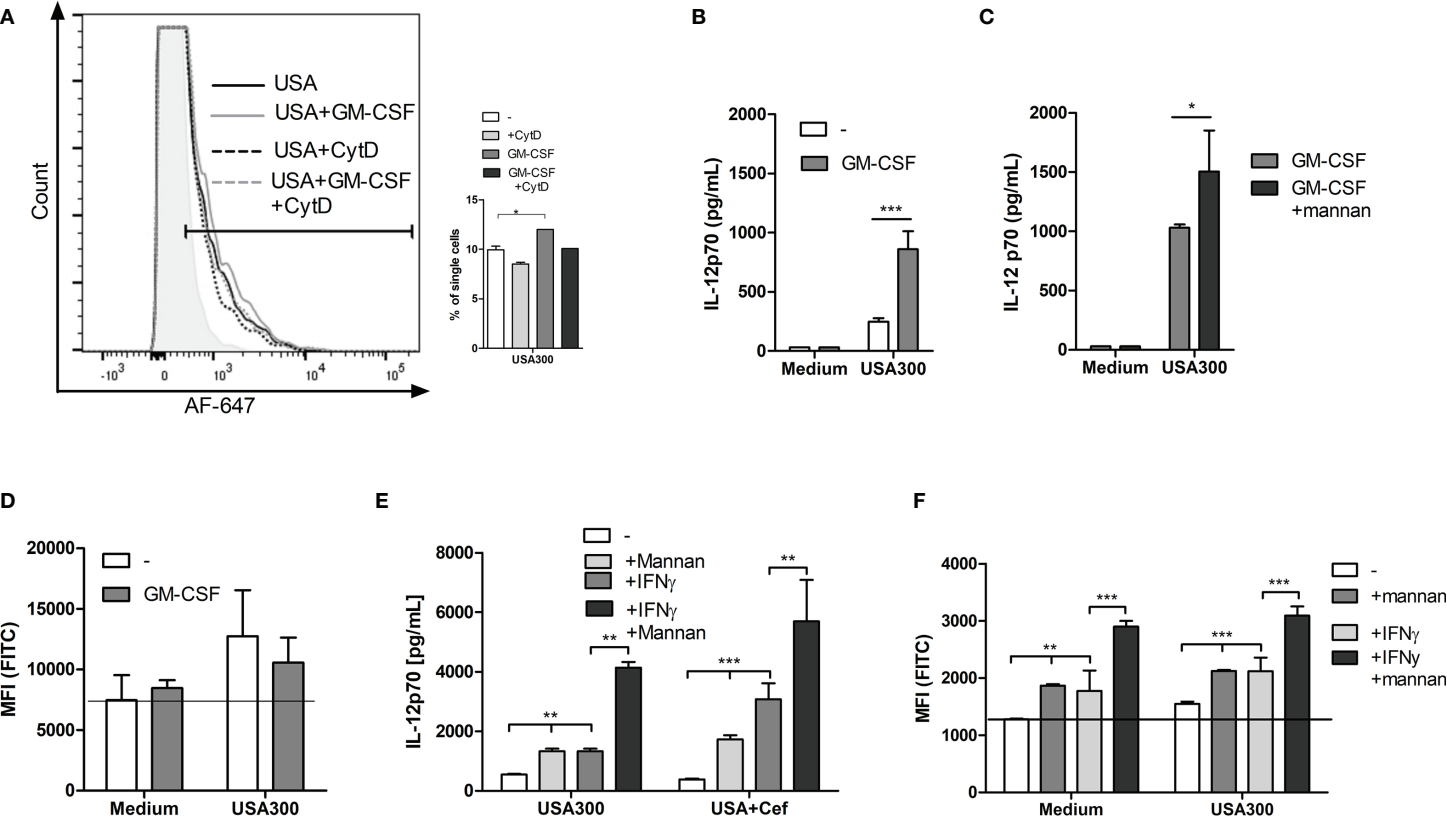
*The IL-12 enhancement by mannan is not affected by inflammatory conditions.* Histograms depict the fluorescence of AF-647-labelled USA300 in DCs pre-treated or not by GM-CSF and with or without CytD treatment. Bars show the number of fluorescent cells after each treatment **(A)**. Cells were treated with GM-CSF and stimulated with USA300 and IL-12 in the supernatant was measured **(B)**. Cells were treated with GM-CSF and mannan before USA300 stimulation and IL12 production was measured **(C)**. DCs were incubated with carboxy-H_2_DCFDA with or without GM-CSF and then stimulated with USA300 for 4 h. The oxidation was measured by flow cytometry, shown as MFI **(D)**. DCs were pre-treated with mannan or not for 30 min prior to USA300 stimulation with IFN-γ in the medium or not. DCs were incubated with carboxy-H_2_DCFDA with or without IFN-γ and then stimulated with USA300 for 4 h. The oxidation was measured by flow cytometry, shown as MFI. For all experiments n=3 +/- SD. 2-way ANOVAs were performed for **(B–F)**, *p < 0.05, **p < 0.01, ***p < 0.001. Data represent one out of at least two experiments.

To investigate whether the effect induced by mannan still had an enhancing effect on IL-12 response and ROS production during inflammatory conditions; DCs were pre-stimulated with GM-CSF or IFN-γ. GM-CSF slightly increased the proportion of DCs taking up AF647-USA300 (**Figure 5A**). Furthermore, the production of IL-12 was enhanced threefold by GM-CSF (**Figure 5B**) and was further enhanced if cells also were pre-treated with mannan (**Figure 5C**). In contrast to mannan, GM-CSF alone or in combination with USA300 did not increase ROS production (**Figure 5D**). Pre-treatment with IFN-γ, able to stimulate maturation and IL-12 production in DCs (24), enhanced the IL-12 production by USA300 to a level corresponding to the level obtained by mannan pre-treatment (**Figure 5D**). In the presence of IFN-γ and mannan, the IL-12 level rose to more than 8 times the level stimulated with USA300 alone and more than three times the level found in the presence of either INF-γ or mannan (**Figure 5E**). While stimulation with USA300 induced a slight ROS induction, the ROS production upon IFN-γ treatment alone corresponded to the level induced by mannan, and, in the presence of both mannan and IFN-γ, the ROS production was further increased both *per se* and in the presence of USA300 (**Figure 5F**).

### Other Ligands and Receptors Employing Clathrin-Mediated Endocytosis May Possess the Same ROS-Stimulating and IFN-β and IL-12-Enhancing Effects

**Figure 6:**
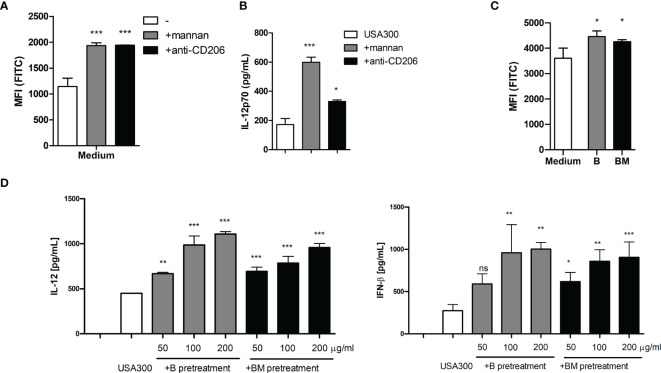
*Anti-MR antibody and soluble β-glucan also enhances MRSA induced IL-12 and ROS production.* Pre-treatment with mannan or with a monoclonal antibody against the mannose receptor (anti-CD206) both induced increased ROS production in DCs **(A)** and enhanced the IL-12 production induced by USA300 **(B)**. Two preparations of soluble β-glucan isolated from barley induced increased ROS **(C)** and enhanced the USA300 induced IL-12 and IFN-β **(D)**. For all experiments n=3 +/- SD. One-way ANOVA, *p < 0.05, **p < 0.01, ***p < 0.001, ns, non significant. All data represent one out of at least two independent experiments.

To investigate whether MR ligation with other soluble molecules would induce ROS production, DCs were incubated with a MR-specific monoclonal antibody (anti-CD206). Adding anti-CD206 increased the ROS production in DCs to a level comparable to that induced by mannan (**Figure 6A**). Furthermore, adding anti-CD206 prior to USA300 stimulation enhanced the USA300-induced IL-12 production (**Figure 6B**). C-type lectin receptors other than MR, e.g. the β-glucan binding receptor dectin-1, also bind and endocytose polysaccharides through clathrin-coated pits (25, 26). Therefore, we investigated whether soluble dectin-1 ligands could induce ROS production and potentiate the MRSA-induced IL-12 response. DCs were exposed to β-glucans, able to bind dectin-1 (25), isolated from two different barley cultivars (19). Both β-glucans induced an increase in the ROS production in unstimulated DCs (**Figure 6C**). Also, the IFN-β and IL-12-enhancing effects of the β-glucans were investigated by pre-treating DCs with increasing concentrations of β-glucan prior to USA300 stimulation. Although no IFN-β or IL-12 was induced by the β-glucans alone, both β-glucans enhanced the USA300-induced IL-12 and IFN-β production in a dose-dependent manner (**Figure 6D**). In summary, other soluble molecules binding to MR and soluble ligands binding to dectin-1 induce ROS production and potentiate the IFN-β an IL-12 production induced by MRSA.

## Discussion

The production of IL-12 by dendritic cells is crucial for clearance of *S. aureus* infections (2, 3). We have previously demonstrated that mannan through MR enhances IL-12 production in DCs stimulated with Gram-positive bacteria but not with Gram-negative bacteria. This enhancement was due to increased bacterial uptake and increased IFN-β production, which in turn led to increased IL-12 production (13). The present study investigated this effect specifically on the response to MRSA and established that the enhancement of IFN-β and IL-12 induction by mannan does not change signaling pathways leading to IFN-β and IL-12 production but merely enhances the uptake of *S. aureus* and increases ROS formation resulting in a more prompt degradation of the endocytosed bacteria.

We demonstrated that mannan potentiates the IFN-β and IL-12 response towards clinical MRSA strains, also if the MRSA was treated with the β-lactam cefoxitin. Importantly, the IL-1β response was not enhanced by mannan indicating that mannan does not generally potentiate an inflammatory response, but merely an anti-bacterial and anti-viral response. We demonstrated that mannan enhanced the endocytosis of bacteria, induced ROS formation, and enhanced the IL-12 and the IFN-β production without changing the major intracellular pathways employed by USA300. Of note, a monoclonal antibody specific for the MR as well as highly purified barley β-glucans previously shown to be internalized through dectin-1 by a clathrin-mediated mechanism (26), similarly induced ROS formation and enhanced IFN-β and IL-12 production induced by MRSA strains. Hence, several C-type lectin receptors such as MR and dectin-1 may share the capacity to upon ligation and endocytosis increase the IL-12 production induced by MRSA probably through enhanced ROS production.

Mannan alone was not able to induce expression or secretion of IL-12, which is in accordance with the lack of an intracellular signaling motif in the MR (27) and the general acceptance of TLR activation as the IL-12 inducing event (28, 29). Still, in the absence of other microbial stimulation mannan induced an appreciable ROS activation corresponding to the level induced by USA300 alone. Suppression of NADPH oxidase by apocynin abrogated the IFN-β and IL-12 production induced by MRSA alone or together with mannan, and the inhibitor of clathrin-coated pits mediated endocytosis (30), MDC, exclusively inhibited the IL-12 and IFN-β production enhanced by mannan. Accordingly, we find it likely that the induced ROS production may be a key factor causing the mannan-enhanced IFN-β and IL-12 production. In addition, mannan increased the uptake and the endosomal killing of the bacteria, the latter a cellular process that also depends on the formation of ROS (31). The ligation of mannan to MR causes internalization of the receptor, which may induce other cellular events including increased immunogenicity of endocytosed matter and cross presentation in DCs (14, 32). The effect of mannan exerted through mannose receptor internalization on ROS formation in DCs has to our knowledge not been reported before, but may very well represent a key event responsible for previous findings on MR’s ability to increase antigen presentation and immunogenicity as ROS formation is a prerequisite for efficient degradation of endocytosed material and the subsequent peptide ligation to MHC molecules (33).

Importantly, the endocytosis of USA300 increased in DCs pre-treated by mannan. In order to induce IFN-β and IL-12 upon stimulation by Gram-positive bacteria, bacteria must be endocytosed as endosomal degradation is crucial (8, 9, 34). Hence, efficient endocytosis as well as a prompt ROS induction represent two mechanisms likely to play key roles in a strong IL-12 induction. Here, we showed that the MR-ligand mannan increased the uptake of USA300 and the resulting IL-12 production by USA300. The induction of IL-12 requires endosomal degradation and the release of TLR ligands that stimulates IL-12 production either directly or indirectly *via* IFN-β induction. The fact that mannose receptor upon internalization ends up in the early endosomes where it leaves its cargo and circulates back into the plasma membrane (27), may indicate that the integration into the endosomal membrane initiates events that lead to acidification of the endosome and thus, the activity of NOX2. IL-12 production is also dependent on phagosome acidification, crucial, not only for lysosomal enzyme activity but also for the activity of NOX2, which consumes protons from the lumen in the process of ROS production (35). The mannan-enhanced ROS production might also indirectly induce IL-12 through an up-regulation of acid sphingomyelinase (ASMase), resulting in increased ceramide production, which in turn leads to increased phagocytosis (36, 37). The released ceramide also directly interacts with, among other proteins, kinase suppressor of Ras, involved in MAP kinase activation (38). In our previous study, we showed that addition of ASMase to DCs increases the uptake of *L. acidophilus* (13). Although the ASMase activity or ceramide formation in response to mannan was not measured here, an increased uptake of USA300 was confirmed in response to mannan pre-treatment. We also demonstrated that mannan up-regulates SLAMF1 expression in DCs (13). In turn, SLAMF1 can induce ROS production through the recruitment of NOX2 subunits to the phagosome (39). This may suggest that SLAMF1 plays a key role in the mannan enhanced ROS production.

We did not formally show which receptor(s) mannan acts through, but we have previously demonstrated that mannan leads to a temporary internalization of MR and reduced uptake of dextran known to be internalized through MR (13). In the present manuscript, we show that a MR specific Mab induces the same effect as mannan on ROS and IL-12 production, which further corroborates the key role of MR in the effect of mannan. This, however, does not exclude that other mannan-binding receptors such as DC-SIGN or its murine equivalent also contribute to the IL-12 potentiating effect. Taken together, these findings strongly indicate that MR is involved in the effects induced by the mannan. Despite this, we did not demonstrate that MR is the sole receptor involved. However, even though we have not formally identified the receptors involved we believe that our findings showing that addition of the soluble carbohydrates to the cells prior to stimulation with S*.aureus* enhances the IFN-β and IL-12 production and induces ROS, is a key finding which may aid in our understanding how specific microbial and plant derived molecules may condition antigen presenting cells.

The mannan enhanced IFN-β and IL-12 production in USA300-stimulated DCs was dependent on bacterial uptake, phagosome acidification, ROS production and signaling through JNK. Induction of IFN-β is dependent on JNK (40) and we have previously shown that endosomal degradation of Gram-positive bacteria leads to induction of IFN-β in turn inducing the induction of IL-12 (11, 13, 34, 41). Hence, rather than changing or inducing a new signaling pathway, mannan enhanced the signals already induced by the bacteria. This suggests that the mannan-induced IL-12 was due to a more potent induction by factors from USA300 rather than from mannan itself. As we found a faster killing of endocytosed MRSA upon mannan pre-treatment, this suggests that the mannan-induced ROS production leads to a faster endosomal release of USA300-derived TLR ligands able to stimulate a stronger and more prompt IL-12 induction.

GM-CSF induces inflammation including increased macropinocytosis in DCs and thus increased uptake of bacteria (42, 43). Accordingly, pre-stimulation with GM-CSF led to increased uptake of USA300 and increased IL-12 production in response to USA300. Mannan also enhanced the USA300-induced IL-12 production in the presence of GM-CSF resulting in even higher IL-12 induction. In contrast to mannan inducing ROS production alone and in combination with USA300, GM-CSF had no effect on ROS production in DCs stimulated with USA300. This demonstrates that different IL-12 stimulatory pathways may act in synergy. Likewise, pre-stimulation with the inflammatory cytokine IFN-γ that stimulates ROS production and bacterial killing in myeloid cells (44–46) led to increased USA300 induced IL-12 production, both in the presence and absence of mannan. Like for mannan, we showed that IFN-γ alone induced ROS production, which might lead to IL-12 induction through increased bacterial degradation, but together with mannan the ROS production was further enhanced. This may imply that mannan and IFN-γ induce ROS production through distinct cellular mechanisms. IFN-γ also induces ROS *via* activation of NOXs but employs another pathway involving JAK/STAT signaling inducing mitochondrial ROS production (42). Notably, IFN-γ is used in experimental setups to prime macrophages and has been reported to augment the production of IL-12 in response to other stimuli (43, 44). Taking together, mannan increased the USA300-induced IL-12 also in the presence of pro-inflammatory cytokines, leading to additionally increased IL-12 production.

We have previously shown that growing MRSA strains including USA300 with a β-lactam shifts the cytokine induction from IL-12 towards IL-23 (20). We demonstrated that in DCs stimulated with cefoxitin-treated USA300 a more prompt *Dusp1* transcription was induced compared to cells stimulated with untreated MRSA. This led to inactivation of JNK and p38 pathways early in the immune cell response. If MR and dectin-1 through mannan and β-glucan, respectively, initiates a strong and prompt ROS production in addition to enhanced endocytosis of MRSA, this may result in an early and stronger JNK signaling leading to IFN-β and IL-12 transcription before the emergence of *Dusp1* and the consequent abrogation of JNK signaling. As the IL-23 transcription seems to be induced by a faster pathway that is independent of JNK, the production of IL-23 is less sensitive to *Dusp1*. Hence, we suggest that an enhanced ROS production may lead to prompt release of TLR ligands in the endosome and thus more rapid and stronger activation of JNK giving rise to more *Ifnb* transcription before JNK is deactivated by DUSP-1.

Taken together our results provide evidence for a Th1-enhancing effect of mannan and β-glucan, two ligands for CTL receptors on antigen presenting cells. Ligation of other CTL receptors may result in similar effects. Thus, CTL receptors may play a key role in regulating the type and strength of the response to pathogens. With the spread of antibiotic resistance, such knowledge may be relevant to exploit in treatment and prevention of pathogen infections.

## Data Availability Statement

The raw data supporting the conclusions of this article will be made available by the authors, without undue reservation.

## Author Contributions

HE and HF designed the research. HE, PJ, and EN performed the majority of the experiments. FJ and ML-S performed the bacteria survival experiments. HI and MB supervised the bacterial experiments. HE and HF analyzed and interpreted data. HE and HF wrote the paper. All authors critically reviewed the manuscript. All authors contributed to the article and approved the submitted version.

## Funding

This work was supported by two grants (Grant DFF-6111-00499 and DFF-1032-00221B) to HF from the Danish Council for independent Research (DFF).

## Conflict of Interest

The authors declare that the research was conducted in the absence of any commercial or financial relationships that could be construed as a potential conflict of interest.

## Publisher’s Note

All claims expressed in this article are solely those of the authors and do not necessarily represent those of their affiliated organizations, or those of the publisher, the editors and the reviewers. Any product that may be evaluated in this article, or claim that may be made by its manufacturer, is not guaranteed or endorsed by the publisher.
